# Iron(II)‐Catalyzed Aerobic Biomimetic Oxidation of Amines using a Hybrid Hydroquinone/Cobalt Catalyst as Electron Transfer Mediator

**DOI:** 10.1002/anie.202102681

**Published:** 2021-05-04

**Authors:** Arnar Guðmundsson, Srimanta Manna, Jan‐E. Bäckvall

**Affiliations:** ^1^ Department of Organic Chemistry Arrhenius Laboratory Stockholm University 10691 Stockholm Sweden; ^2^ Department of Natural Sciences Mid Sweden University 85170 Sundsvall Sweden

**Keywords:** aerobic oxidation, amines, electron transfer, homogeneous catalysis, iron

## Abstract

Herein we report the first Fe^II^‐catalyzed aerobic biomimetic oxidation of amines. This oxidation reaction involves several electron transfer steps and is inspired by biological oxidation in the respiratory chain. The electron transfer from the amine to molecular oxygen is aided by two coupled catalytic redox systems, which lower the energy barrier and improve the selectivity of the oxidation reaction. An iron hydrogen transfer complex was utilized as the substrate‐selective dehydrogenation catalyst along with a bifunctional hydroquinone/cobalt Schiff base complex as a hybrid electron transfer mediator. Various primary and secondary amines were oxidized in air to their corresponding aldimines or ketimines in good to excellent yield.

## Graphical abstract

An iron(II)‐catalyzed aerobic biomimetic oxidation of amines has been developed. The principle is based on biological oxidation in the respiratory chain in living organisms. The electron transfer from the amine to molecular oxygen occurs through coupled redox reactions, mediated by a bifunctional hydroquinone/cobalt Schiff base as hybrid catalyst. This hybrid catalyst was found to significantly shield the iron catalyst from deactivation.

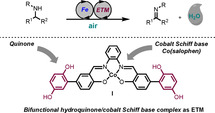

Oxidation processes constitute an important fundamental class of transformations in organic chemistry. Although numerous oxidation reactions have been developed over the years, the demand for milder, more efficient, and sustainable methods has increased in recent times with the growing interest in green chemical procedures.^[1]^ With regard to green methods, of particular interest are those inspired by biological processes,[^2^, ^3^] where environmentally friendly and inexpensive oxidants such as molecular oxygen (O_2_) or hydrogen peroxide (H_2_O_2_) are often used. However, direct selective oxidation of an organic substrate by H_2_O_2_ or O_2_ remains an unmet challenge because of the large energy barriers and low selectivity of such direct oxidations. The use of a substrate‐selective redox catalyst (SSRC) may solve this problem, where the reduced form of the SSRC (i.e. SSRC_red_) is re‐oxidized by H_2_O_2_ or O_2_. However, direct re‐oxidation of the SSRC_red_ to SSRC by O_2_ or H_2_O_2_ may still be too slow and there are only a limited number of examples known in the literature of direct reoxidation of an SSRC_red_ by O_2_ or H_2_O_2_.[^2^, ^3^] In Nature this oxidation problem is solved by enlisting multiple enzymes and co‐enzymes as electron transfer mediators (ETMs), which lower the overall barrier for electron transfer from the SSRC_red_ to H_2_O_2_ or O_2_ as shown in Scheme 1. In natural aerobic systems, these ETMs are part of the respiratory chain, which is responsible for producing ATP in many organisms. The overall process of the respiratory chain is analogous to the process shown in Scheme 1 and ends with reduction of O_2_ to H_2_O.^[4]^ Over the years we have developed a number of biomimetic oxidations that work according to the principle shown in Scheme 1, where ruthenium,^[5]^ palladium,^[6]^ and osmium,^[7]^ have been used as substrate‐selective redox catalysts.^[8]^

**Scheme 1:**
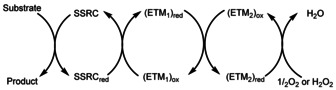
Principle for oxidation with O_2_ or H_2_O_2_ using ETMs (ETM=electron transfer mediator; SSRC=substrate selective redox catalyst).

Recently the groups of Beller and Bolm declared that the age of iron had begun, and it is certainly true that the field has advanced rapidly since then.^[9]^ Over the past two decades, inexpensive iron catalysts have been employed in many elegant synthetic transformations which have traditionally been dominated by noble transition metal catalysts, such as cross‐coupling and transfer hydrogenation, among many others.[^10^, ^11^, ^12^] We have recently developed iron‐catalyzed reactions including DKR of *sec*‐alcohols, cycloisomerization of functionalized allenes, and biomimetic aerobic oxidation of alcohols.[^13^, ^14^] In the present work we have developed a novel iron‐catalyzed aerobic oxidation of amines via the biomimetic approach in Scheme 1, where ETM_1_ and ETM_2_ are merged into the bifunctional ETM **I**, which acts as a hybrid catalyst (Scheme 2).

**Scheme 2:**
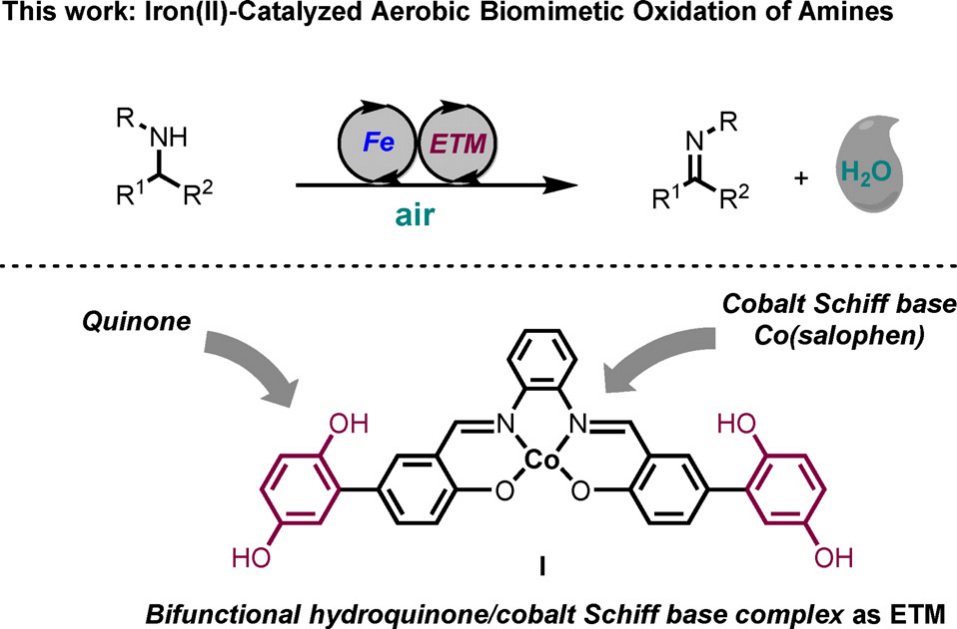
Iron catalyzed biomimetic oxidation of amines using cobalt hybrid hydroquinone catalyst **I**.

A prominent class of iron catalysts that have been used for transfer hydrogenation are the (cyclopentadienone)iron tricarbonyl complexes **II**, originally synthesized by Reppe and Vetter in the 1950′s (Scheme 3).^[15]^ Iron hydride complex **III** was first prepared and isolated by the group of Knölker.[^16^, ^17^] Knölker's complex **III** and its related iron tricarbonyl complexes **II** have found extensive use in transfer hydrogenation reactions.[^18^, ^19^] The first use of complex **III** in catalysis was reported by the group of Casey in 2007 for the hydrogenation of ketones.^[19]^ Later, the group of Funk reported the DMPh (DMPh=3,5‐dimethylphenyl) tricarbonyl variant **IIa** of these complexes,^[18a]^ which was found to be more active in redox reactions than all other tricarbonyl complexes **II** tested. Complex **IIa** can be activated in situ to generate intermediate **IIa′**, which in turn can be reduced to iron hydride **IV** in the presence of a hydrogen source (Scheme 3).^[20]^ The activation of the catalyst (**IIa**→**IIa′**) is done through oxidative decarbonylation induced by trimethylamine *N*‐oxide (TMANO).^[21]^ Our group has recently applied these types of iron complexes in several iron‐catalyzed reactions.[^13^, ^14^]

**Scheme 3:**
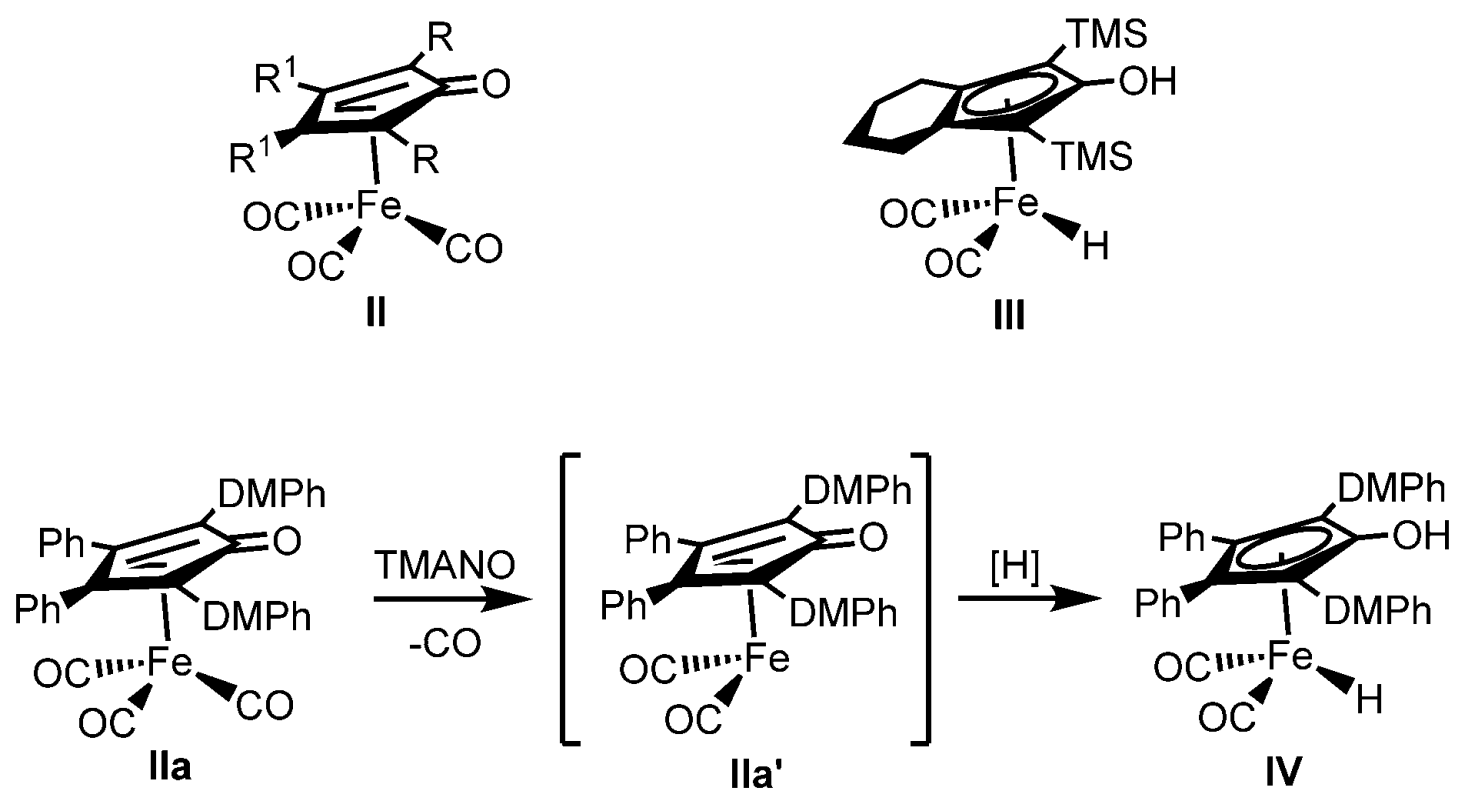
Activation of iron tricarbonyl complex **IIa** and Knölker*’s* complex (**III**).

Recently we developed an iron‐catalyzed biomimetic oxidation of alcohols employing two electron transfer mediators, 2,6‐dimethoxy benzoquinone (DMBQ) and the cobalt Schiff‐base catalyst Co(salmdpt).^[13a]^ In that study alcohol **1** afforded ketone **2** in good yields in the aerobic oxidation. After the completion of that study, we envisioned that a similar iron‐catalyzed biomimetic oxidation of amine substrates by molecular oxygen might be feasible, in particular since the corresponding ruthenium‐catalyzed aerobic oxidation of amines had previously been realized with the same ETMs.^[5b]^ Surprisingly, attempts to use these two ETMs (DMBQ and Co(salmdpt)) for the oxidation of **3 a** were unsuccessful and only trace amounts of imine **4 a** were obtained (Scheme 4). Apparently, the electron transfer to molecular oxygen is too slow in this case, resulting in predominating competing deactivation of the iron catalyst by molecular oxygen.

**Scheme 4:**
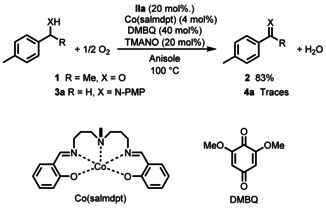
Previous aerobic oxidation of alcohol **1** to ketone **2** and attempted oxidation of amine **3** **a** to imine using Co(salmdpt) and DMBQ as separate ETMs.

One way to circumvent this problem would be to increase the rate of the electron transfer from the iron catalyst to molecular oxygen. In previous studies we have obtained a faster electron transfer in oxidations by merging the two ETMs (the Co‐Schiff base and the quinone) into a bifunctional ETM, which acts as a hybrid catalyst.[^22^, ^23^] Interestingly, using such a bifunctional ETM (hybrid catalyst **I** in Scheme 2) in place of two separate ETMs (cf. Scheme 4) in the oxidation of **3 a** to **4 a** gave a 24 % yield after 2 h (Table 1, entry 1). The iron‐catalyzed biomimetic oxidation of PMP‐protected benzylamine **3 a** (PMP=*p*‐methoxyphenyl) as model substrate was run in anisole as solvent using 10 mol % of iron catalyst **IIa**, TMANO, and hybrid catalyst **I** under air. Iron(II) complexes such as **II′**, **III** and **IV** are known to be extremely sensitive to air and although the DMPh iron catalyst variant is more resistant to air than other analogues of its type, the main drawback in our earlier work was the fact that after 2 h the DMPh iron catalyst was found to be completely deactivated. Surprisingly, when using hybrid catalyst **I** in entry 1, full conversion was observed after running the reaction overnight, meaning that the presence of **I** somehow suppresses oxidative deactivation of the iron catalyst. The reason for this decreased oxidative degradation is not clear, but may be due to a more efficient quenching of O_2_ by **I** and a more efficient reoxidation of **IV** to **IIa′**. This allows for a much longer reaction time, which is essential for the more electron‐deficient (slower reacting) substrates, making the protocol broader in scope. Other solvents than anisole were screened as well (entries 2–11), and it was found that toluene and 1,4‐dioxane gave the best results (entries 2 and 5). We next lowered the temperature and found that MeOH as solvent at 40 °C gave an even better result (entry 12) than dioxane at 80 °C (cf. entry 5). It is plausible that the cause of this improvement is the redox activity of MeOH, which could aid the overall oxidative process. Running the reaction at 60 °C in MeOH allowed for a lowering of the catalyst loading of **IIa** to 5 mol % (entry 13). The optimal conditions were found to be a 1:1 mixture of MeOH and dioxane at 60 °C and under these conditions **4 a** was obtained in 73 % NMR yield after 2 h and in excellent yield (>95 %) after 16 h (entry 14).

**Table 1:** Optimization of reaction conditions.^[a]^ 
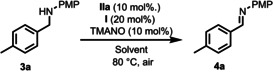

Entry^[a]^	Solvent	NMR Yield 1 h [%]^[b]^	NMR Yield 2 h [%]^[b]^
1	Anisole	8	24	
2	Toluene	18	25	
3	CPME	4	7	
4	2‐Me THF	9	13	
5	1,4‐Dioxane	21	40	
6	DCE	6	6	
7	DMSO	9	18	
8	DMF	3	8	
9	1‐Propanol	10	19	
10	H_2_O	0	0	
11	Pentadecane	3	3	
12^[c]^	MeOH	20	41	
13^[d]^	MeOH	26	69	
14^[d,f]^	MeOH/1,4‐Dioxane	25	73 (>95^[e]^)	

[a] General reaction conditions: The reaction was conducted at 80 °C with 0.25 mmol of **3 a**, 0.025 mmol of **I**, 0.05 mmol of **IIa**, 0.025 mmol of TMANO, and 2 mL of solvent. [b] Yields were determined by NMR analysis using 1,3,5‐trimethoxybenzene. [c] Reaction was performed at 40 °C. [d] 5 mol % of **IIa**, 5 mol % of TMANO and 10 mol % of **I** were used at 60 °C. [e] NMR yield after 16 h. [f] MeOH and Dioxane were used in a 1:1 ratio. DCE=1,2‐dichloroethane.

With the fully optimized reaction conditions in hand, we next turned our attention to investigating the substrate scope of various benzylamine derivatives (Table 2). Electron‐donating and electron‐withdrawing substituents on the benzylamine had little effect on the reaction and products **4 a**–**4 f** were obtained in good to excellent yields (entries 1–6, Table 2). It should be noted that the *p*‐MeO‐substituted benzylamine **3 b**, was fully converted to **4 b** (>95 % NMR yield) but the isolated yield was only 82 % due to decomposition on purification (entry 2). Introducing a more sterically hindered *o*‐methyl substituent on the benzylamine caused a drop in conversion but as expected the desired product **4 f** was stable and could be isolated in 68 % yield (entry 6). Naphtyl‐substituted product **4 g** was also obtained in an excellent yield (entry 7). 2‐Furylmethylamine derivative **3 h** was well tolerated and aldimine product **4 h** was isolated in 60 % yield (entry 8). Pyrrolidine substrate **3 i** could be oxidized to the corresponding ketimine in 56 % isolated yield (entry 9). It became apparent that the substituent on the nitrogen is crucial for the success of the reaction, with the electron‐rich PMP group giving the best result. Changing this group to Ph led to a significant loss in conversion with only 25 % NMR yield of **4 j** being observed (entry 10). Amines with other protecting groups such as Ms, Ts or *tert*‐butyl were all unreactive. Amines bearing a methyl group at the R^2^‐position were also tested but were found to give complex mixtures. Presumably oxidation of the α‐proton occurs in these cases, resulting in highly reactive enamines. A notable exception to this limitation, however, is the oxidation of **3 i** to product **4 i** (entry 9).

**Table 2:** Substrate scope. 
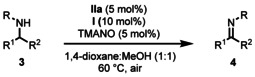

Entry^[a]^	Substrate	Product	Yield [%]
1	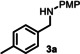	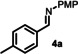	95^[a]^ (>95^[b]^)
2	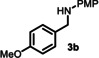	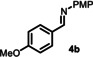	82 (>95^[b]^)
3	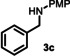	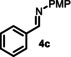	85^[a]^ (85^[b]^)
4	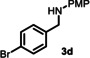	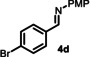	83^[a]^ (87^[b]^)
5	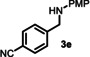	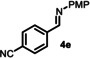	89^[a]^ (91^[b]^)
6	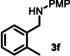	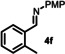	68^[a]^ (73^[b]^)
7	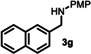	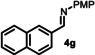	90^[a]^ (95^[b]^)
8	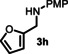	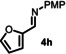	60^[a]^ (70^[b]^)
9	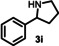	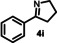	56^[a]^ (95^[b]^)
10	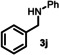	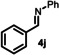	(25^[b]^)

Substrate scope. General reaction conditions: The reaction was conducted under air at 60 °C for 16 h with 0.25 mmol of **3**, 0.0125 mmol of **IIa**, 0.0125 mmol of TMANO, 0.025 mmol of **I**, 1 mL of 1,4‐dioxane and 1 mL of MeOH. [a] Isolated yields. [b] NMR yield determined by using 1,3,5‐trimethoxybenzene as internal standard.

Based on our results, a plausible mechanism is proposed in Scheme 5. The initially activated iron complex **IIa′** is formed by reaction with TMANO via oxidative decarbonylation as shown in Scheme 3. After that, the active catalyst species **IIa′** reacts with substrate **3** to generate **4** and hydride intermediate **IV**.^[24]^ Intermediate **IV** is then oxidized by the benzoquinone fragments of the oxidized hybrid catalyst (**VII**), resulting in the regeneration of **IIa′** and **I**. Reaction of **I** with molecular oxygen results in the generation of Co^III^‐superoxide adduct **V**. Intramolecular electron transfer from one of the hydroquinone fragments to Co with concomitant proton abstraction produces Co‐quinone species **VI** and water. A second intramolecular electron transfer from hydroquinone to Co together with a proton transfer gives **VII** and water. Further investigations into the nature of the mechanism are underway in our laboratory.

**Scheme 5:**
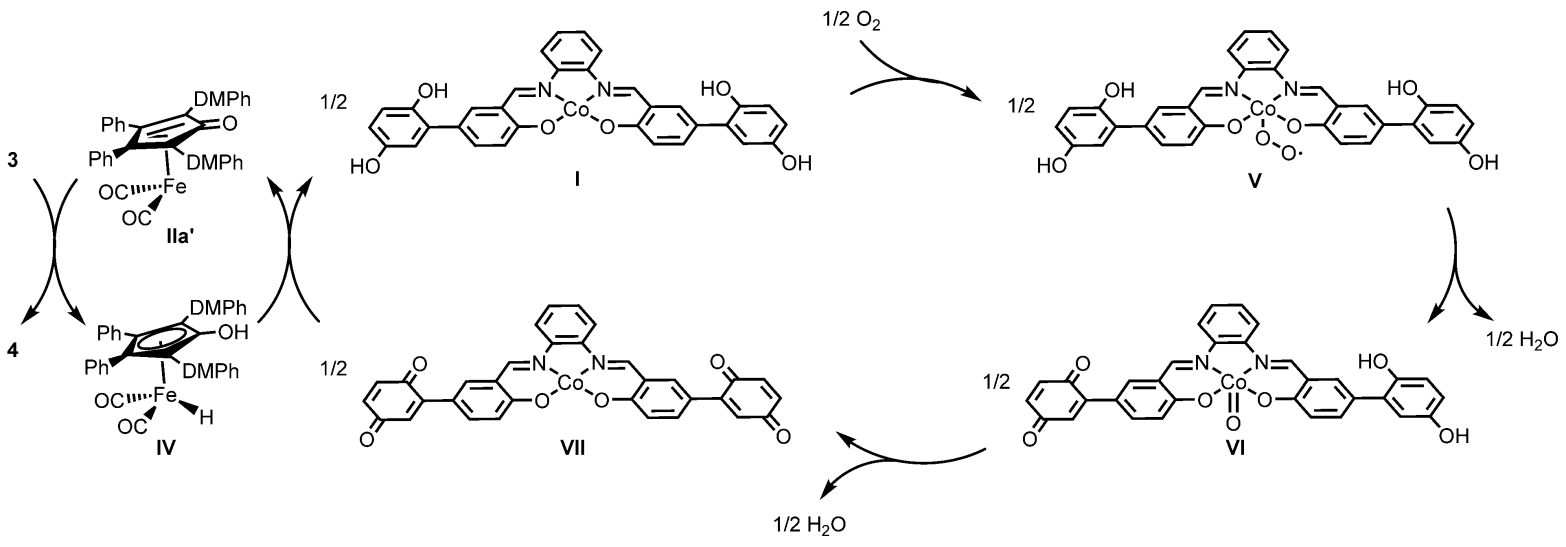
The biomimetic oxidation approach using iron catalyst **IIa** as the SSRC and hybrid hydroquinone/cobalt catalyst **I** as ETM.

Although the mechanism of the electron transfer from the hydroquinone in **I** to molecular oxygen has not been studied in detail, a similar mechanism for electron transfer‐mediated reactions has been studied by the group of Stahl where cobalt(salophen) and hydroquinone were used as separate ETMs.^[25]^ In this study it was proposed that the hydroquinone interacts with the Co^III^‐superoxide intermediate (cf. **V** in Scheme 5) via hydrogen bonding, finally leading to a proton coupled electron transfer. This mechanism seems less likely with the bifunctional hybrid catalyst **I**, where an intramolecular electron transfer coupled with proton transfer probably occurs.

In conclusion, we have developed the first biomimetic oxidation of amines using an iron(II) catalyst together with a bifunctional hybrid catalyst. A hybrid hydroquinone/cobalt Schiff base was used as the ETM and was unexpectedly found to extend the lifetime of the iron catalyst and protect it from oxidative deactivation. The electron transfer system is reminiscent of that occurring in the respiratory chain (Fe catalyst vs. NADH and hybrid catalyst **I** vs. the ubiquinone‐cytochrome c system). Various amines were oxidized to their corresponding imines in good to excellent yields using this biologically inspired method.

## Conflict of interest

The authors declare no conflict of interest.

## Supporting information

As a service to our authors and readers, this journal provides supporting information supplied by the authors. Such materials are peer reviewed and may be re‐organized for online delivery, but are not copy‐edited or typeset. Technical support issues arising from supporting information (other than missing files) should be addressed to the authors.

Supplementary
