# Supplementary material for: Iron(II)‐Catalyzed Aerobic Biomimetic Oxidation of Amines using a Hybrid Hydroquinone/Cobalt Catalyst as Electron Transfer Mediator
Source: Angew Chem Int Ed Engl. 2021 May 4;60(21):11819–23. doi: 10.1002/anie.202102681 (PMC8252094; doi:10.1002/anie.202102681)
Supplement: Supplementary file 1 — Supplementary [file ANIE-60-11819-s001.pdf]

Angewandte Chemie
Eine Zeitschrift der Gesellschaft Deutscher Chemiker

Supporting Information

Iron(II)-Catalyzed Aerobic Biomimetic Oxidation of Amines using a Hybrid Hydroquinone/Cobalt Catalyst as Electron Transfer Mediator

*Arnar Guðmundsson[+], Srimanta Manna[+], and Jan-E. Bäckvall**

anie_202102681_sm_miscellaneous_information.pdf

Arnar Guðmundsson,† Srimanta Manna† and Jan-E. Bäckvall*

†Arnar Guðmundsson and Srimanta Manna contributed equally to this work

E-mail: jeb@organ.su.se

Table of Contents:

1. General experimental details S2
2. Spectroscopic data S5
3. References S13

1. General experimental details

General information

^{1}H NMR and ^{13}C NMR spectral data were recorded at 400 MHz and 100 MHz, respectively. Chemical shifts (δ) are reported in ppm, using the residual solvent peak in $CDCl_3$ (H = 7.26 and C = 77.0 ppm) as internal standard, and coupling constants (*J*) are given in Hz. HRMS were recorded using ESI-TOF techniques. Iron complex **IIa** was synthesized using an established procedure.[1] Chemicals were purchased from commercial sources and dried before use. Silica gel chromatography was performed manually (particle size 40-63µm, pore size 60Å and mesh size 230-400) using silica that had been neutralized with trimethylamine. Reactions were monitored using aluminum-backed plates (1.5 Å, 5 cm) pre-coated (0.25 mm) with silica gel, UV light or potassium permanganate stain for visualization.

General procedure for the iron(II)-catalyzed biomimetic oxidation

To a flame dried and inert 25 mL Schlenk flask was added **3** (0.25 mmol), **I** (14.7 mg, 0.025 mmol), **IIa** (7.5 mg, 0.0125 mmol), TMANO (1 mg, 0.0125 mmol), dry 1,4-dioxane (1 mL) and dry MeOH (1 mL). The Schlenk flask was fitted with an air-filled balloon and stirred at 60 °C for 16 h to give imine **4**. Upon completion, the solvent was removed *in vacuo* and the crude product was purified by column chromatography.

(*E*)-*N*-(4-Methoxyphenyl)-1-(*p*-tolyl)methanimine (4a)

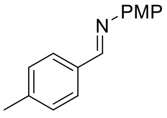

4a

4a was prepared according to the general procedure for iron(II)-catalyzed biomimetic oxidation. Eluent system for column chromatography: n-pentane/EtOAc 100/0 - 10/90 (v/v). Spectral data is consistent with data reported in the literature.[2] Isolated yield: 95 %.

^{1}H NMR (400 MHz, Acetone) δ ppm 8.56 (s, 1H), 7.87 – 7.73 (m, 2H), 7.34 – 7.28 (m, 2H), 7.30 – 7.23 (m, 2H), 7.01 – 6.92 (m, 2H), 3.82 (s, 3H), 2.39 (s, 3H).

***N*,1-Bis(4-methoxyphenyl) methanimine (4b)**

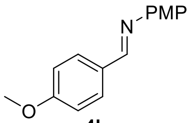

4b

4b was prepared according to the general procedure for iron(II)-catalyzed biomimetic oxidation. Eluent system for column chromatography: n-pentane/EtOAc 100/0 - 10/90 (v/v). Spectral data is consistent with data reported in the literature.[3] Isolated yield: 82 %

^{1}H NMR (400 MHz, CDCl$_3$) δ ppm 8.41 (s, 1H), 7.89 – 7.74 (m, 2H), 7.23 – 7.18 (m, 2H), 6.99 – 6.96 (m, 2H), 6.95 – 6.90 (m, 2H), 3.87 (s, 3H), 3.83 (s, 3H).

(*E*)-*N*-(4-Methoxyphenyl)-1-phenylmethanimine (4c)

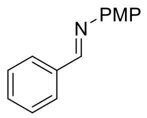

4c

4c was prepared according to the general procedure for iron(II)-catalyzed biomimetic oxidation. Eluent system for column chromatography: n-pentane/EtOAc 100/0 - 10/90 (v/v). Spectral data is consistent with data reported in the literature.[2]

^{1}H NMR (CDCl$_3$, 400 MHz) δ ppm 8.52 (s, 1H), 7.88-7.96 (m, 2H), 7.47-7.54 (m, 2H), 7.27 (d, 2H, *J* = 8.6 *Hz*), 6.97 (d, 2H, *J* = 8.6 *Hz*), 3.86 (s, 3H). Isolated yield: 83 %.

(*E*)-*N*-(4-Methoxyphenyl)-1-(*p*-bromo)methanimine (4d)

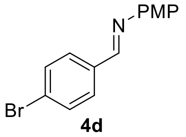

4d

4d was prepared according to the general procedure for iron(II)-catalyzed biomimetic oxidation. Eluent system for column chromatography: n-pentane/EtOAc 100/0 - 10/90 (v/v). Spectral data is consistent with data reported in the literature.[3] Isolated yield: 83 %.

^{1}H NMR (400 MHz, Acetone) δ ppm 8.62 (s, 1H), 7.89 (d, *J* = 8.5 *Hz*, 2H), 7.69 (d, *J* = 8.5 *Hz*, 2H), 7.31 (d, *J* = 8.9 *Hz*, 2H), 6.98 (d, *J* = 8.9 *Hz,* 2H), 3.82 (s, 3H).

(*E*)-4-(((Methoxyphenyl)imino)methyl)benzonitrile (4e)

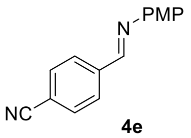

4e

4e was prepared according to the general procedure for iron(II)-catalyzed biomimetic oxidation. Eluent system for column chromatography: n-pentane/EtOAc 100/0 - 10/90 (v/v). Spectral data is consistent with data reported in the literature.[4] Isolated yield: 89 %.

^{1}H NMR (CDCl$_3$, 400 MHz) δ ppm 8.52 (s, 1H), 7.99 (d, 2H, *J* = 8.6 *Hz*), 7.74 (d, 2H, *J* = 8.6 *Hz*), 7.28 (d, 2H, *J* = 8.8 *Hz*), 6.95 (d, 2H, *J* = 8.8 *Hz*), 3.85 (s, 3H).

(*E*)-*N*-(4-Methoxyphenyl)-1-(*o*-tolyl)methanimine (4f)

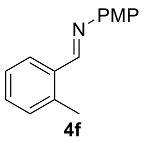

4f

4f was prepared according to the general procedure for iron(II)-catalyzed biomimetic oxidation. Eluent system for column chromatography: n-pentane/EtOAc 100/0 - 10/90 (v/v). Spectral data is consistent with data reported in the literature.[3] Isolated yield: 68 %.

^{1}H NMR (CDCl$_3$, 400 MHz) δ ppm 8.77 (s, 1H), 8.06 (d, *J* = 7.4 *Hz,* 1H), 7.27-7.37 (m, 2H), 7.22 (d, *J* = 8.9 *Hz,* 2H), 6.94 (d, *J* = 8.9 *Hz,* 2H), 3.84 (s, 3H), 2.59 (s, 3H).

(*E*)-N-(4-Methoxyphenyl)-1-(naphthalen-2-yl)methanimine (4g)

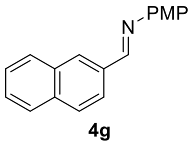

4g

4f was prepared according to the general procedure for iron(II)-catalyzed biomimetic oxidation. Eluent system for column chromatography: n-pentane/EtOAc 98/2 - 10/90 (v/v). Spectral data is consistent with data

reported in the literature.[5] Isolated yield: 90 %.

1H NMR (400 MHz, CDCl3) δ ppm 8.65 (s, 1H), 8.21 – 8.14 (m, 2H), 7.97 – 7.84 (m, 3H), 7.58 – 7.49 (m, 2H), 7.34 – 7.27 (m, 2H), 7.02 – 6.86 (m, 2H), 3.85 (s, 3H)

(*E*)-1-(Furan-2-yl)-*N*-(4-methoxyphenyl)methaneimine (4h)

4h was prepared according to the general procedure for iron(II)-catalyzed biomimetic oxidation. Eluent system for column chromatography: n-pentane/EtOAc 98/2 - 15/85 (v/v). Spectral data is consistent with data reported in the literature.[6] Isolated yield: 60 %.

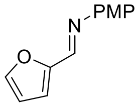

4h

^{1}H NMR (400 MHz, CDCl$_3$) δ ppm 8.33 (s, 1H), 7.61 (d, *J* = 1.7 *Hz*, 1H), 7.34 – 7.20 (m, 2H), 7.05 – 6.85 (m, 2H), 6.56 (dd, *J* = 3.5, 1.8 *Hz*, 1H), 3.85 (s, 3H).

5-Phenyl-3,4-dihydro-2H-pyrrole (4i)

4i was prepared according to the general procedure for iron(II)-catalyzed biomimetic oxidation. Eluent system for column chromatography: n-pentane/EtOAc 100/0 - 10/90 (v/v). Spectral data is consistent with data reported in the literature.[7] Isolated yield: 56 %.

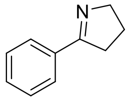

4i

^{1}H NMR (400 MHz, CDCl$_3$) δ ppm 7.88 – 7.81 (m, 2H), 7.41 (dd, *J* = 5.5, 1.8 Hz, 3H), 4.07 (tt, *J* = 7.4, 2.0 Hz, 2H), 2.96 (ddt, *J* = 8.3, 7.3, 2.0 Hz, 2H), 2.04 (dq, *J* = 9.0, 7.5 Hz, 2H)

(*E*)-*N*,1-Diphenylmethanimine (4j)

4j was prepared according to the general procedure for iron(II)-catalyzed biomimetic oxidation. Eluent system for column chromatography: n-pentane/EtOAc 100/0 - 10/90 (v/v). Spectral data is consistent with data reported in the literature.[2] NMR yield as determined using 1,3,5-trimethoxybenzene as internal standard: 25 %.

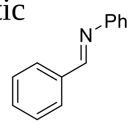

4j

2. Spectroscopic data

^{1}H NMR spectrum of **4a**

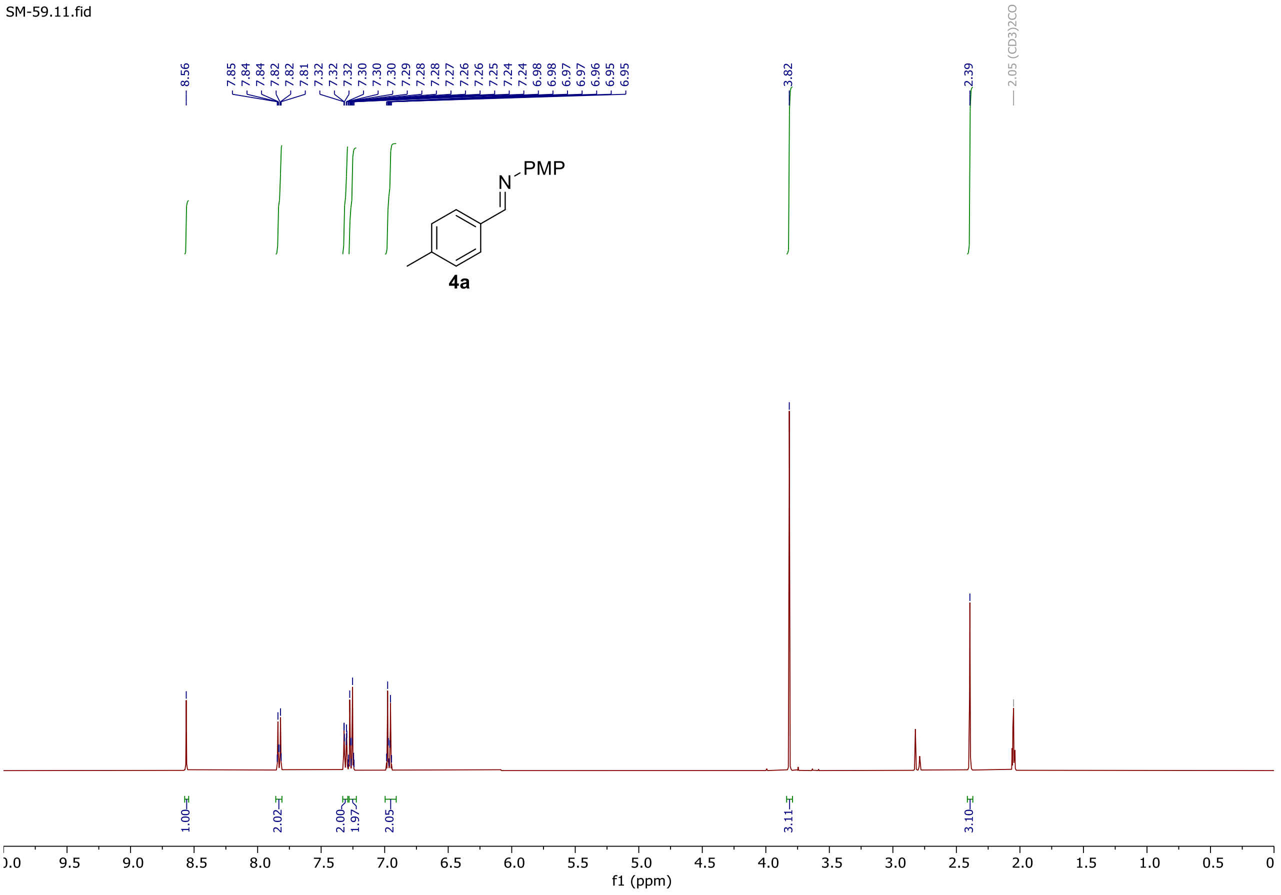

^{1}H NMR spectrum of **4b**

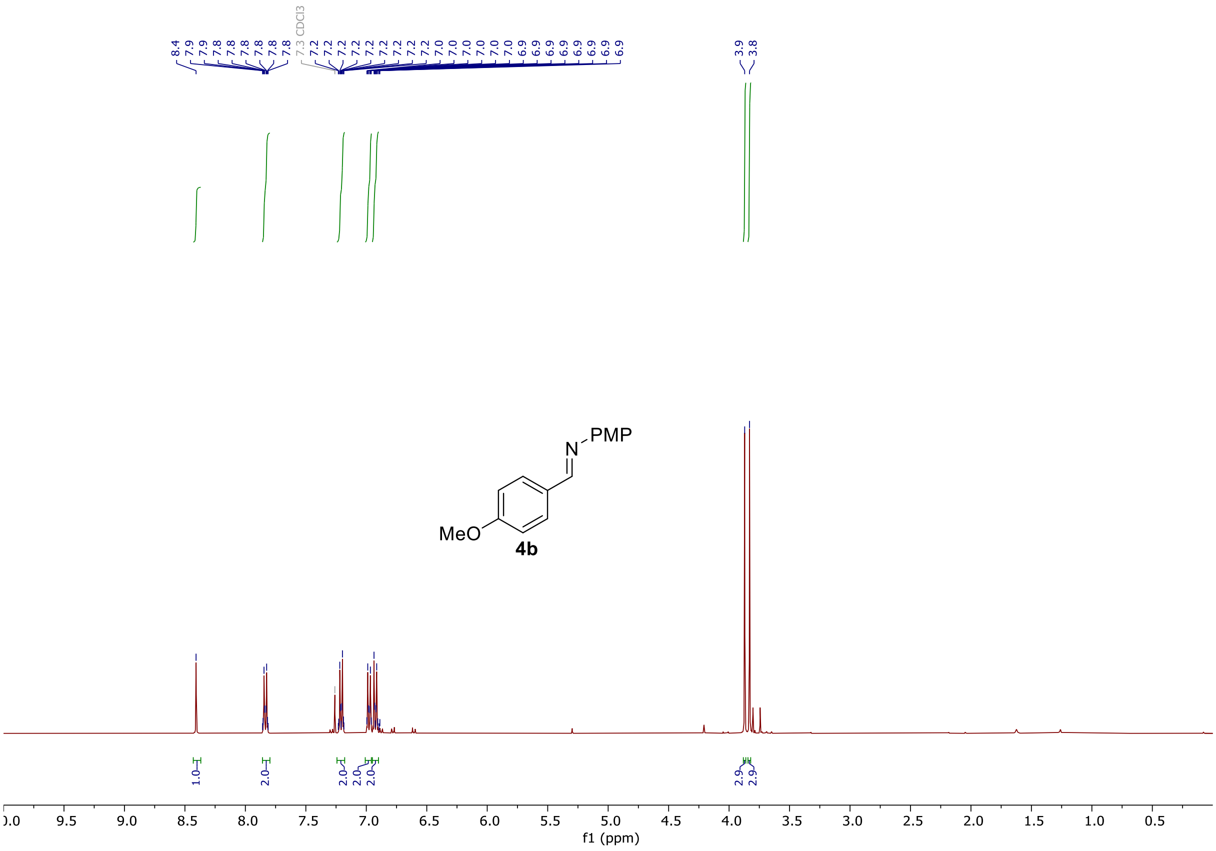

^{1}H NMR spectrum of **4c**

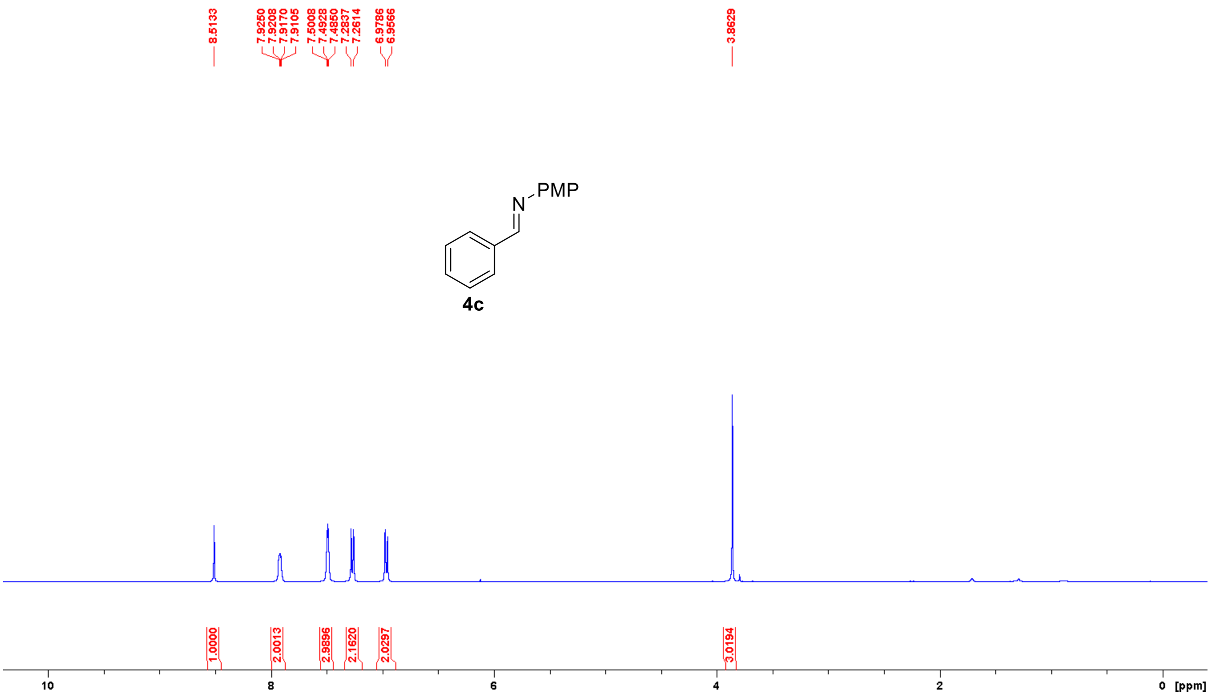

^{1}H NMR spectrum of **4d**

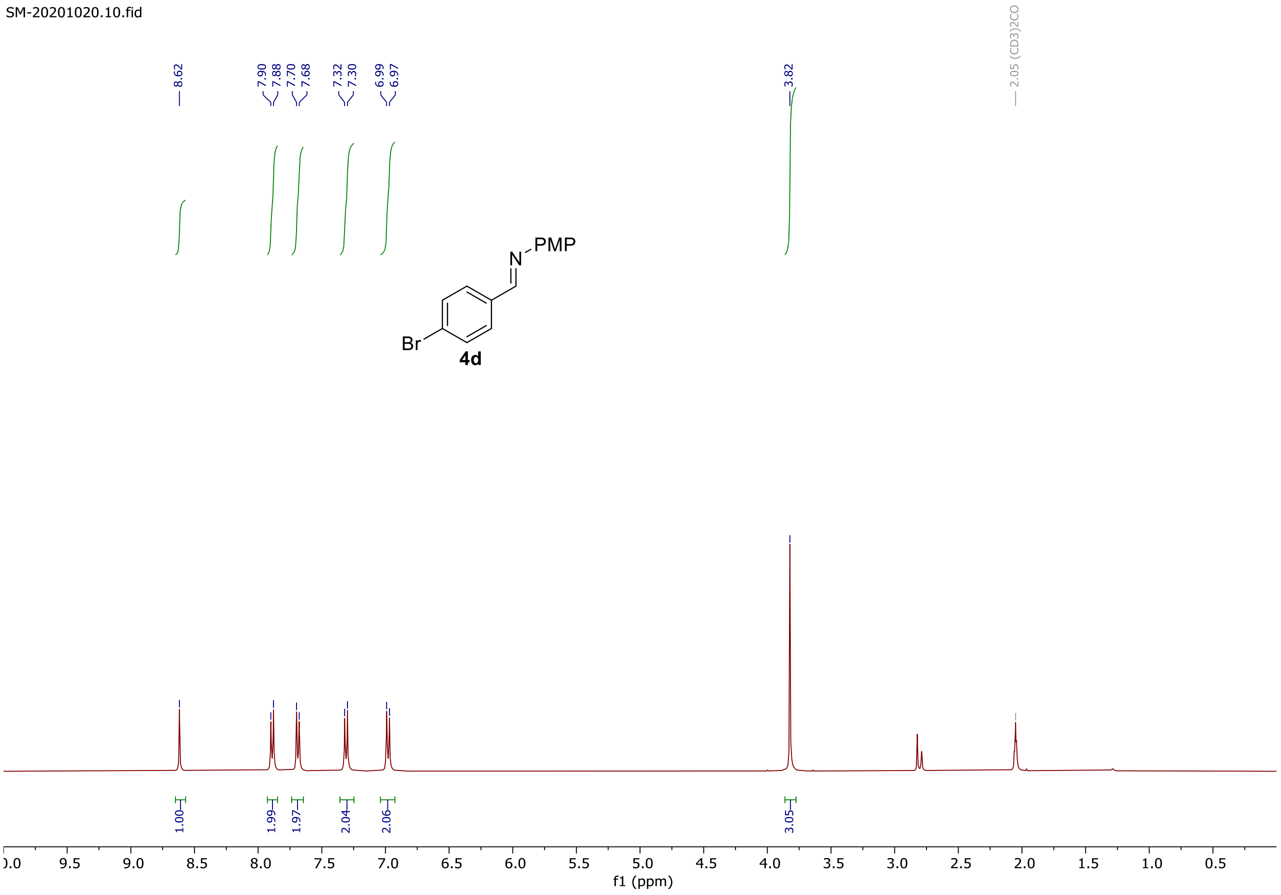

^{1}H NMR spectrum of **4e**

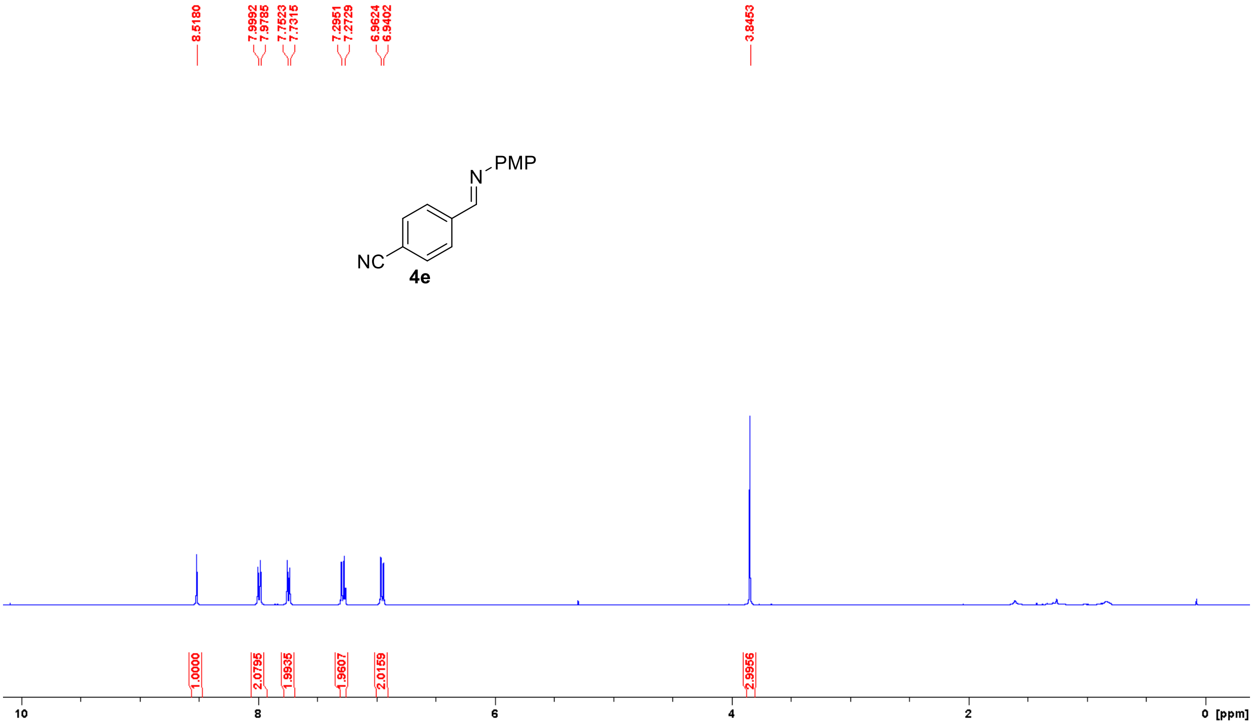

^{1}H NMR spectrum of **4f**

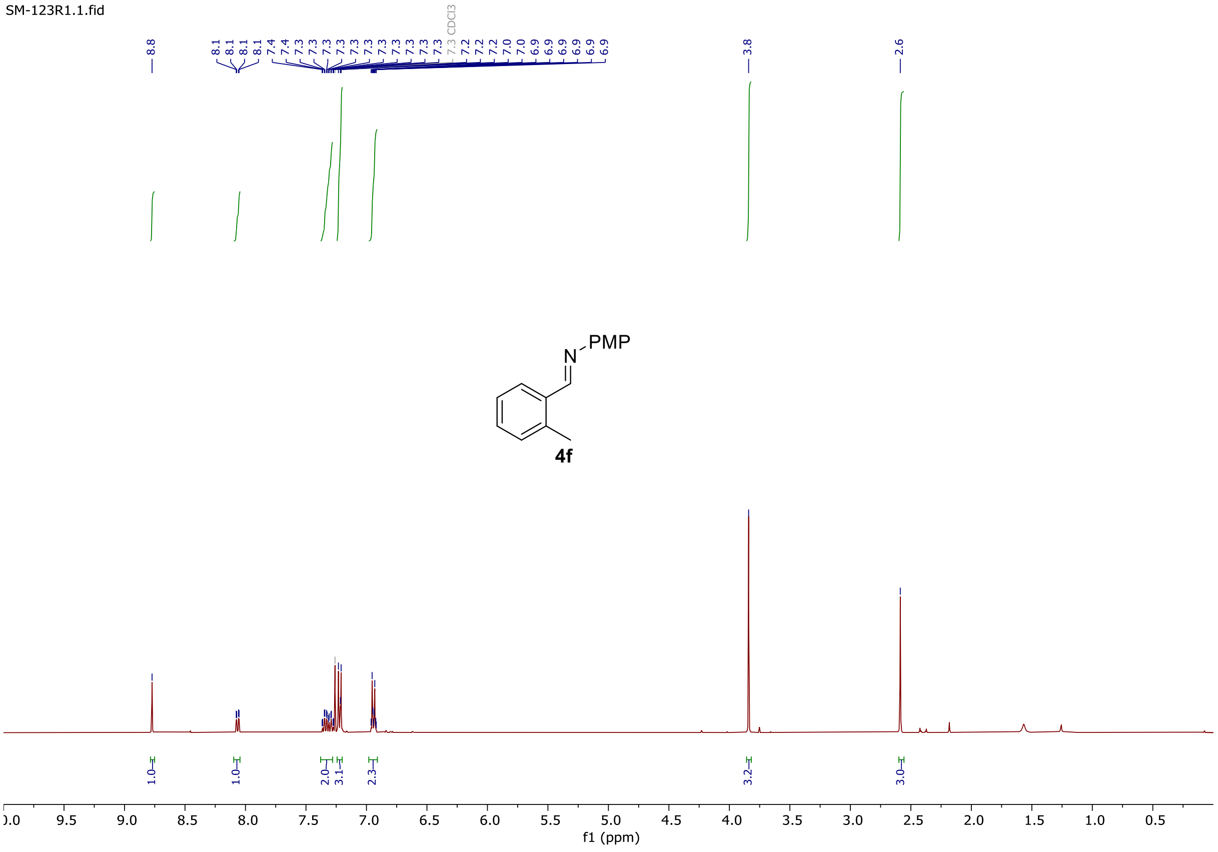

^{1}H NMR spectrum of **4g**

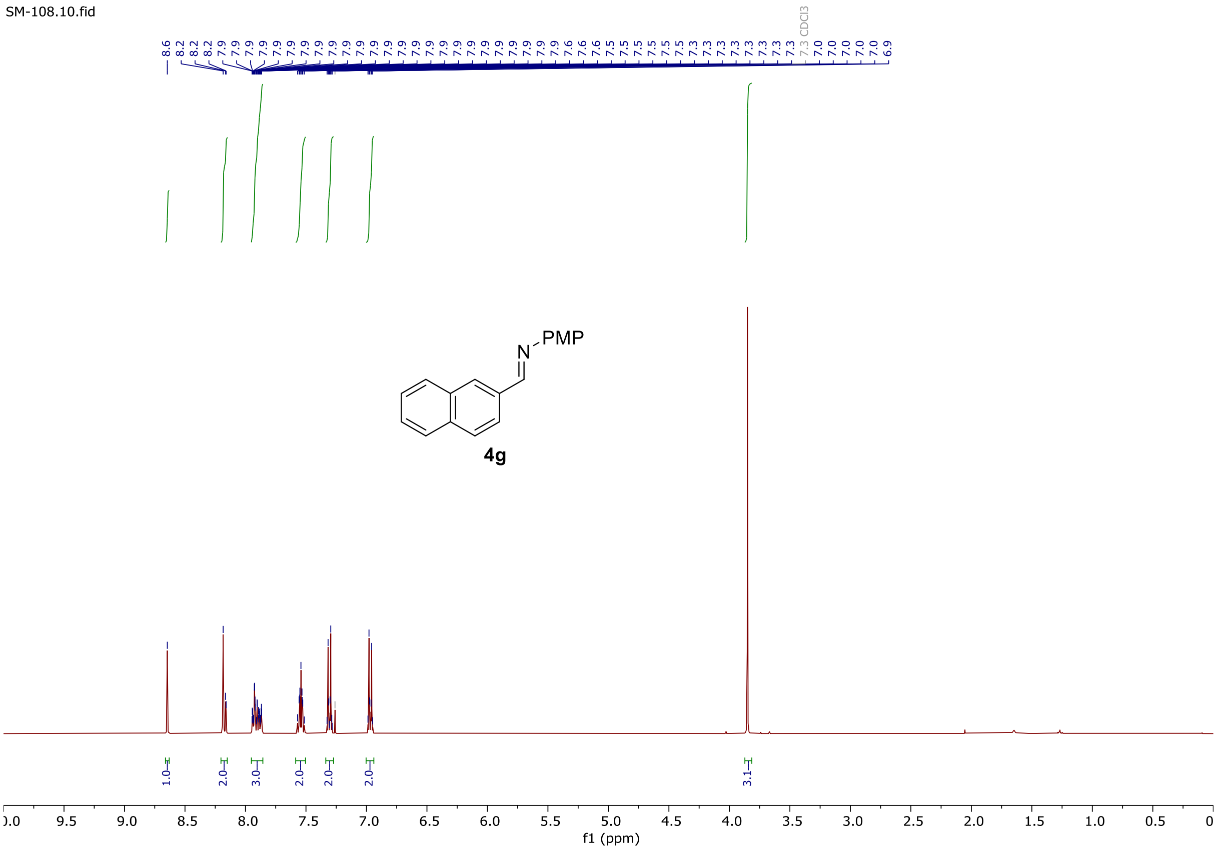

^{1}H NMR spectrum of **4h**

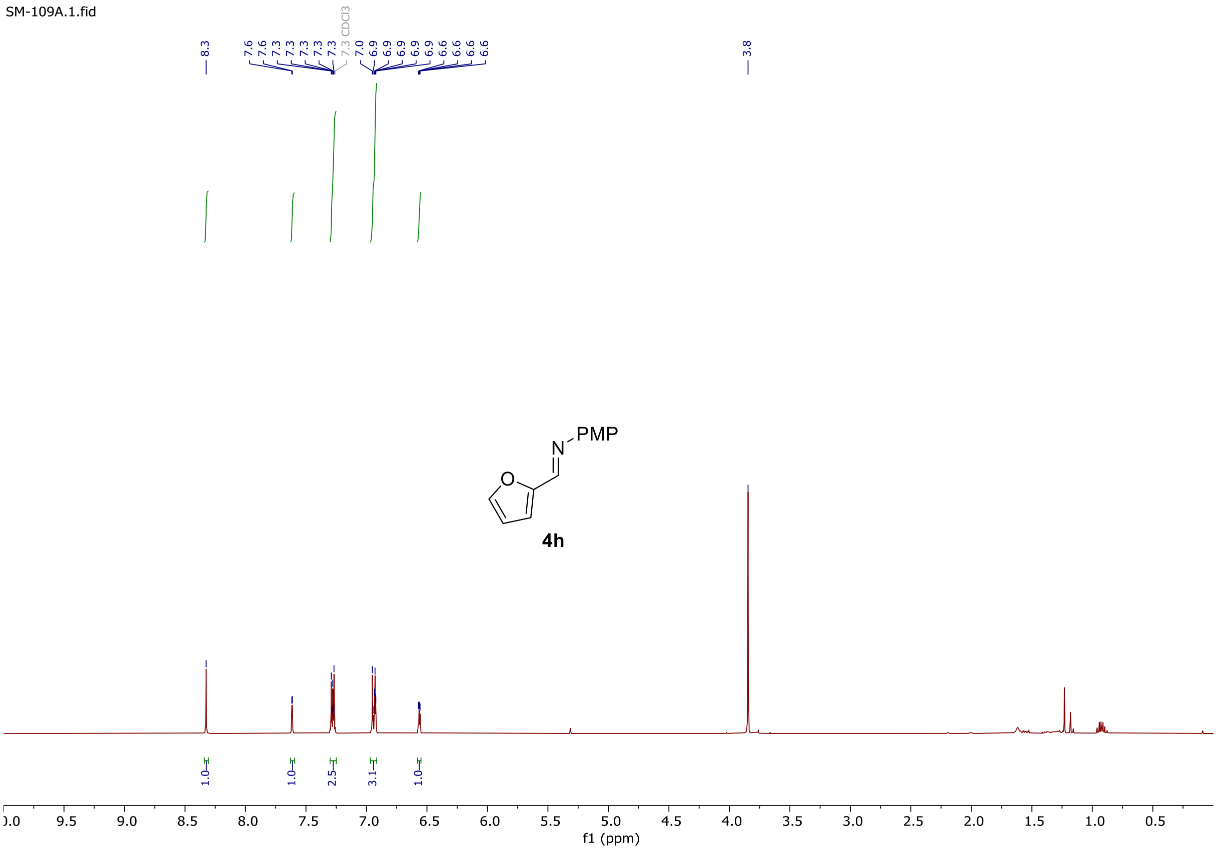

^{1}H NMR spectrum of **4i**

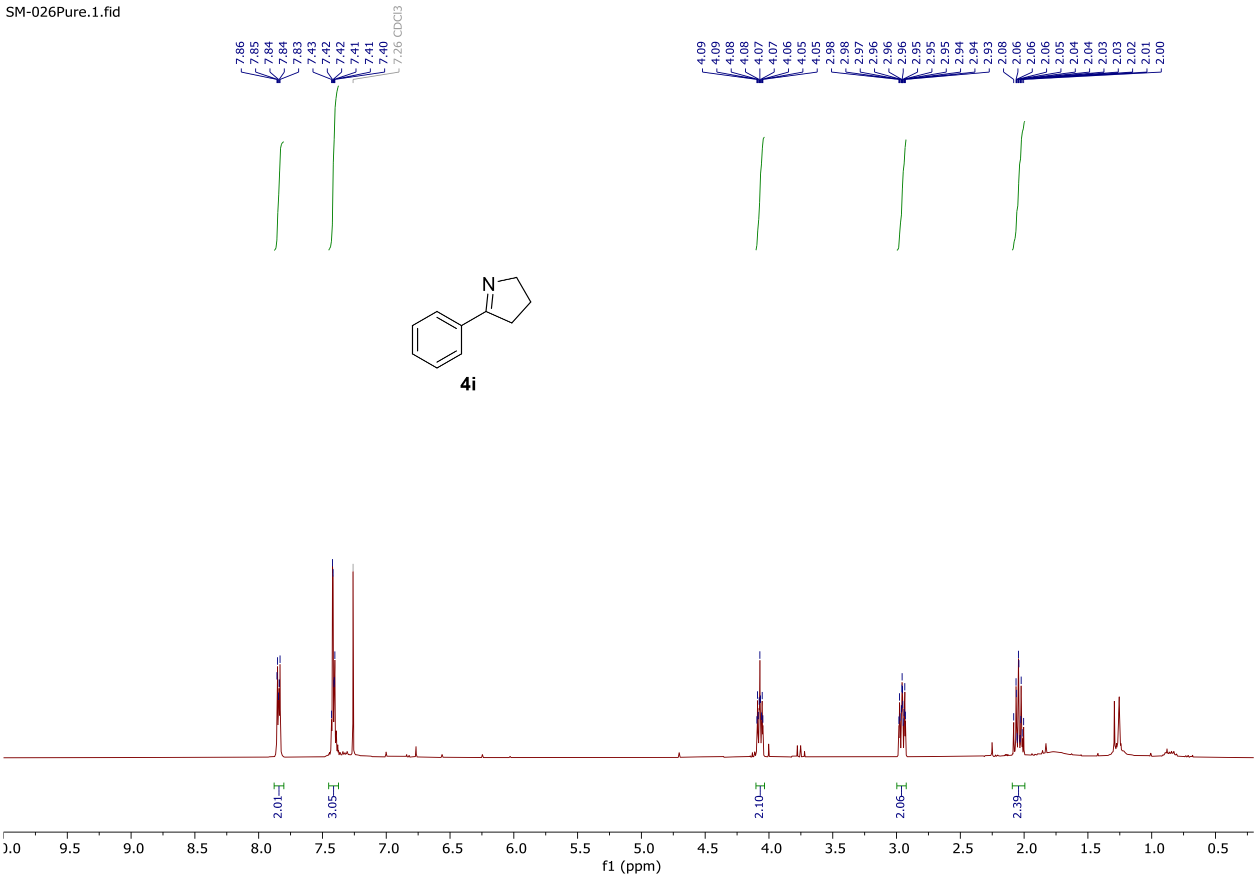

3. References

(1) T. W. Funk, A. R. Mahoney, R. A. Sponenburg, P. Z. Kathryn, D. K. Kim, E. E. Harrison. *Organometallics* **2018**, *37*, 1133-1140.

(2) B. Guo, H-X. Li, S-Q. Zhang, D. J. Young, J-P. Lang. *ChemCatChem* **2018**, *10*, 5627-5636.

(3) M. G. Kallitsakis, P. D. Tancini, M. Dixit, G. Mpourmpakis, I. N. Lykakis *J. Org. Chem.* **2018**, *83*, 1176-1184.

(4) A. Kumar, A. G. Samuelson *J. Organomet. Chem.* **2010**, *695*, 338-345.

(5) Y.-F. Zhang, B. Wu, Z.-J. Shi, *Chem. Eur. J.* **2016**, *22*, 17808-17812.

(6) Q. Jiang, J.-Y. Wang, C. Guo, *J. Org. Chem.* **2014**, *79*, 8768-8773.

(7) F. Huang, S. Zhang, *Org. Lett.* **2019**, *21*, 7430–7434.
